# The Light and Shadow of Smartphone Applications for Eating Disorders: Commentary on Cruz et al. (2025)

**DOI:** 10.1002/eat.24536

**Published:** 2025-09-09

**Authors:** Silvia Cerea

**Affiliations:** ^1^ Department of General Psychology University of Padova Padova Italy

**Keywords:** eating disorders, flourishing, randomized controlled trial, smartphone apps, treatment

## Abstract

Smartphone applications (apps) represent promising tools to overcome common barriers to treatment in individuals within the Eating Disorders (EDs) spectrum, thanks to their constant availability and cost‐effectiveness. In this context, Cruz et al. (2025) conducted the first meta‐analysis of randomized controlled trials (RCTs) evaluating the efficacy of app‐based interventions for EDs. Their findings provided evidence supporting the use of app‐based interventions over control conditions in reducing ED symptoms and risk factors, although effect sizes were generally small. Despite these promising findings, caution is needed due to methodological concerns in the included trials, such as limited sample diversity, inconsistent follow‐up durations, and the frequent use of waitlist controls. Notably, no significant effects were observed for key ED dimensions such as body dissatisfaction/disturbance and drive for thinness, pointing to the need for interventions grounded in robust body image theory and frameworks. Furthermore, the current evidence does not adequately address the efficacy of these apps across different populations, including adolescents, gender‐diverse individuals, and those from varied racial, ethnic, and socioeconomic backgrounds. To maximize the clinical utility of these tools, future research should prioritize methodologically rigorous trials, adopt inclusive and intersectional frameworks, and expand evaluative outcomes beyond symptom reduction to include multiple indicators of psychological flourishing. These directions are essential to unlock the full potential of app‐based interventions for EDs across diverse populations.

**Summary**

- This commentary outlines points for reflection, major challenges, and future directions for the use of app‐based interventions in eating disorders.
- Main limitations concern methodological weaknesses, the gap between app availability and empirical evidence, and the narrow demographics of study participants.
- The absence of significant effects on body dissatisfaction highlights the need for apps to more effectively address body image and related risk factors.
- Future research should adopt more rigorous designs, prioritize inclusivity, and evaluate outcomes beyond symptom reduction, including psychological flourishing.

## 1 Introduction

While evidence‐based therapies for eating disorders (EDs) do exist, many individuals suffering from or at risk of these conditions lack access to such interventions or do not receive adequate care. Common barriers to treatment include the high cost associated with interventions, geographic distance from mental health services, perceived stigma and shame, and lack of anonymity.

Smartphone applications (apps) may help overcome these barriers by offering accessible and cost‐effective interventions, ensuring anonymity and providing constant availability (Linardon et al. 2020). Numerous apps for ED treatment are currently available on major app stores (e.g., iOS and Android). However, their clinical utility remains unclear, as the majority lack empirical evaluation of their efficacy through rigorous studies such as randomized controlled trials (RCTs).

The meta‐analysis by Cruz et al. (2025) is the first study to systematically assess the efficacy of app‐based interventions for EDs. By synthesizing data from 14 RCTs, findings revealed small but significant effects for most of the outcomes in samples without formal diagnoses and when apps were used as self‐help interventions. Exceptions were found for body dissatisfaction/disturbance and drive for thinness, where no significant effects emerged. In formally diagnosed samples, evidence was limited to objective binge eating in individuals with a diagnosis of bulimia nervosa or binge‐eating disorder. Overall, medium‐sized effects for objective binge eating emerged when apps were incorporated into guided self‐help. Less consistent results emerged when apps were used as adjuncts to traditional therapy.

The study by Cruz et al. (2025) offered invaluable points for reflection and future research in the field, allowing clinicians and researchers to deepen their understanding of the utility of app‐based interventions in EDs psychopathology, both as self‐help tools and as adjuncts to traditional therapy. While the findings are encouraging, Cruz et al. (2025) also raised several concerns. They suggest caution when considering these apps as fully suitable, particularly for individuals with a formal ED diagnosis. This cautious stance is warranted in light of the following points for reflection and major challenges.

## 2 Points for Reflection and Major Challenges

First, a key point for reflection concerns the limited number of RCTs included in the meta‐analysis (i.e., 14 studies), which makes the promising findings still preliminary. In addition, methodological concerns were identified in 8 of the 14 studies included in the meta‐analysis, particularly regarding random sequence generation, allocation concealment, and incomplete outcome data. These methodological weaknesses raise important questions about the strength of the conclusions that can be drawn. Moreover, most of the studies used a waitlist control group as the comparator, a design choice that complicates the interpretation of the results. Indeed, the use of a waitlist control group makes it difficult to determine whether observed improvements are attributable to the specific therapeutic components of the apps or rather to nonspecific factors, such as the digital placebo effect or participant expectations. Taken together, these limitations highlight the need for more robust and methodologically rigorous RCTs to confirm the preliminary evidence of the efficacy of app‐based interventions for EDs.

Building on this, a second major limitation relates to the generalizability of the findings. Most participants in the reviewed studies were white, highly educated women over the age of 18. This narrow demographic profile makes it difficult to draw conclusions about the efficacy of app‐based interventions for individuals with different characteristics, such as adolescents, a group widely recognized as being at heightened risk for the development of EDs due to factors such as body dissatisfaction, social media exposure, and identity development challenges.

In parallel, the efficacy of app‐based interventions for EDs should also be examined among marginalized groups, including individuals with diverse gender identities, ethnicities, races, and socioeconomic backgrounds. App‐based interventions may be especially beneficial for these groups, as they have the potential to overcome many of the barriers to treatment that disproportionately affect these populations by providing anonymity, reducing stigma and geographic barriers, and delivering scalable and low‐cost interventions. Their cost‐effectiveness is particularly relevant for individuals with low socioeconomic status, who often face limited access to ED‐specific services due to financial constraints, geographic barriers, or systemic inequities. By reducing the need for in‐person appointments, these interventions have the potential to bridge gaps in care and provide timely, flexible support to those who might otherwise remain untreated. Moreover, for individuals outside the skinny, white, affluent, girls profile, who are often underrepresented in research and under‐identified in clinical practice (Sonneville and Lipson 2018), these interventions may be particularly valuable, helping to counteract the stigma that can hinder help‐seeking. By focusing almost exclusively on populations already disproportionately identified and treated for EDs, current research risks missing the opportunity to fully capture the potential benefits of app‐based interventions for marginalized groups. However, expanding demographic representation alone may not be sufficient to fully understand how these interventions function across groups, as a more nuanced analytical lens is required. Applying an intersectionality framework, which considers how multiple social identities (e.g., age, gender, socioeconomic status) interact to shape individuals' lived experiences, can offer deeper insight into variation in the efficacy of app‐based interventions. This perspective is particularly relevant in the context of EDs, where stigma, help‐seeking behaviors, and symptom presentation often differ across intersecting identities.

Third, another important point for reflection concerns the absence of significant effects on body dissatisfaction/disturbance or drive for thinness. This is particularly concerning, as body dissatisfaction is one of the most well‐established and robust risk factors for the development and maintenance of ED symptoms (Stice 2002). These findings raise important questions about whether current app‐based interventions are adequately designed to target body image. Future research should focus on identifying and testing evidence‐based techniques that specifically target body image in the context of EDs. Promising approaches include media literacy, embodiment and intuitive eating practices, and functionality appreciation‐based activities, all of which could be adapted for digital delivery. Moreover, the development of app‐based interventions grounded in a strong theoretical framework of body image (e.g., objectification theory, tripartite influence model, embodiment theory) may significantly enhance the clinical relevance and efficacy of these tools and ensure that all key dimensions of ED psychopathology are properly addressed.

Lastly, while the development of app‐based interventions for EDs is rapidly expanding, a critical gap persists between availability and evidence. Cruz et al. (2025) pointed out that, although many apps targeting EDs are currently available for download in app stores, only a small number (*n* = 9) have been evaluated in RCTs. This is a significant concern, as it raises the risk that users may engage with interventions that are untested, ineffective, or potentially harmful. It is therefore crucial to ensure that only evidence‐based apps are promoted and made accessible to users. In parallel, efforts should be made to replicate and validate existing findings in order to strengthen the empirical foundation of app‐based interventions and better inform clinicians, users, and the broader public about their clinical utility.

## 3 Future Directions

To confirm the preliminary evidence of the efficacy of app‐based interventions for EDs reported by Cruz et al. (2025), future studies should aim to overcome the major challenges highlighted above, moving beyond methodological limitations and evaluating broader outcomes such as flourishing.

Firstly, future studies in the field would benefit from greater methodological rigor, particularly by reducing the current over‐reliance on waitlist control groups. The inclusion of well‐chosen active comparators is crucial to disentangle the unique effects of app‐based interventions from non‐specific factors. In this regard, psychoeducational modules providing evidence‐based information without structured cognitive and behavioral change techniques could represent an optimal active comparator. Such controls can help determine whether improvements are due to the intervention's therapeutic strategies rather than general informational content. Additional possibilities include generic well‐being or relaxation apps that match the intervention in user interaction and design features, while lacking the specific therapeutic strategies under investigation. Considering and explicitly incorporating these alternatives in future research will not only strengthen the interpretability of results but also enhance the clinical relevance and real‐world applicability of app‐based interventions for EDs.

At the same time, considering the chronic nature of EDs, future research should prioritize the inclusion of long‐term follow‐ups to evaluate the maintenance of intervention effects and better understand the potential of app‐based interventions to provide lasting change, since the meta‐analysis by Cruz et al. (2025) was unable to draw firm conclusions, largely due to substantial variability in follow‐up durations across studies (i.e., from 16 days to 2 years in samples without a formal ED diagnosis, and from 3 to 9 months in samples with a formal diagnosis).

Secondly, future research should adopt intersectional approaches and prioritize inclusivity, both in the development and evaluation of the efficacy of app‐based interventions for EDs. This framework can provide a more nuanced understanding of how these apps function across diverse groups, ultimately promoting greater equity in access and outcomes. To address existing gaps, future studies should implement targeted recruitment strategies to engage underrepresented and marginalized groups, including partnerships with community organizations, schools, clinics, and online platforms that reach diverse demographic segments. Additionally, social media outreach can help recruit participants who may not access traditional research settings. Moreover, culturally and contextually adapted contents, such as tailored language, imagery, examples, and intervention features, can enhance interventions relevance and acceptability. Measures to facilitate engagement, including co‐design workshops, feedback loops, or advisory panels involving members from marginalized communities, can ensure usability and sustained participation. Finally, applying intersectional analyses to study how multiple identities interact to influence engagement, adherence, and outcomes will further clarify how these interventions work across populations. By integrating inclusive designs, targeted recruitment, and intersectional analysis, future research can maximize both the reach and the efficacy of app‐based interventions for EDs, ensuring that their benefits extend beyond the populations most commonly studied to those who may need them the most.

Third, future work should aim to reduce the gap between the availability of app‐based interventions for EDs and the empirical evidence supporting their efficacy. Beyond concerns about efficacy, there is a need for transparency: users seeking support for EDs often lack clear guidance on which apps are evidence‐based and which may be ineffective or potentially harmful. To address these challenges, strategies should focus on ensuring that only empirically validated apps for EDs are promoted accessible. One potential approach is the creation of centralized, curated registries of empirically validated apps, clearly highlighting the therapeutic components they target (e.g., media literacy, emotion regulation, cognitive restructuring) and their demonstrated efficacy. Complementary to this, standardized labeling or certification systems could serve as a “seal of approval,” providing users and clinicians with clear information about app efficacy, target populations, and intervention techniques. Policy and regulatory initiatives will be critical to implement these systems. Potential actions include providing guidance to app stores, requiring disclosure of empirical evidence, or incentivizing developers to conduct rigorous evaluations before public release. Given the global availability of many apps, differences in policy, regulatory frameworks, and healthcare integration across countries must be considered, as these factors influence accessibility, promotion, and the evidence required for dissemination. Finally, integrating evidence‐based apps into clinical practice, for instance as part of stepped‐care models, can help ensure that users are directed toward interventions with demonstrated efficacy, maximizing both safety and clinical utility.

A final and equally important area for future research concerns whether app‐based interventions can promote individuals' flourishing beyond the mere reduction of ED psychopathology. The meta‐analysis by Cruz et al. (2025) primarily focused on ED risk factors and symptom reduction, leaving unexplored the potential impact of app‐based interventions on broader dimensions of well‐being. This aspect is crucial, as our goal as researchers and clinicians is not just to reduce symptoms but to promote psychological flourishing (Csikszentmihalyi and Seligman 2000). In the context of EDs, flourishing includes fostering positive body image, enhancing self‐esteem, promoting intuitive and flexible eating, improving quality of life and social functioning, and reducing comorbid symptoms such as anxiety and depression, which are prevalent in ED populations and may be particularly elevated among marginalized or low socioeconomic status groups. These outcomes are not secondary but crucial components of recovery. Despite the growing availability of app‐based interventions for EDs, few studies have explicitly evaluated their effects on broader dimensions of well‐being and comorbid anxiety and depressive symptoms. Addressing this gap is thus a key priority for future research, helping to ensure that app‐based interventions for EDs provide holistic benefits beyond reducing ED symptoms.

## 4 Conclusions

App‐based interventions for EDs have the potential to offer scalable and cost‐effective solutions, with added benefits such as increased accessibility and constant availability. The meta‐analysis by Cruz et al. (2025) contributed to providing evidence favoring app‐based interventions in reducing ED symptoms and risk factors, although the effect sizes were generally small. Despite such promising findings, important challenges remain, particularly concerning methodological rigor, sample representativeness, and the absence of effects of these apps on key ED dimensions, including body dissatisfaction. Addressing these challenges requires a dual focus. On one hand, future research should strengthen methodological rigor through more robust study designs, longer follow‐ups, active comparators, and diverse, representative samples, including adolescents and marginalized groups. On the other hand, it is essential to advance our theoretical and practical understanding of how these interventions work, for whom, and under which conditions. Incorporating insights from existing theoretical models and applying intersectional frameworks will help improve engagement and efficacy across populations. Equally important is the need to broaden the evaluative lens beyond symptom reduction, toward fostering positive outcomes such as body and functionality appreciation, self‐esteem, psychological well‐being, and reduction of comorbid symptoms like anxiety and depression. Applying these strategies will be essential for realizing the full potential of app‐based interventions for ED psychopathology, ensuring they are not only evidence‐based but also inclusive, person‐centered, and clinically meaningful across diverse populations.

## Author Contributions

**Silvia Cerea:** conceptualization, writing – original draft, writing – review and editing.

## Conflicts of Interest

The author declares no conflicts of interest.

## Data Availability

Data sharing not applicable to this article as no datasets were generated or analysed during the current study.
